# Slight crack identification of cottonseed using air-coupled ultrasound with sound to image encoding

**DOI:** 10.3389/fpls.2022.956636

**Published:** 2022-09-15

**Authors:** Chi Zhang, Wenqian Huang, Xiaoting Liang, Xin He, Xi Tian, Liping Chen, Qingyan Wang

**Affiliations:** ^1^Intelligent Equipment Research Center, Beijing Academy of Agriculture and Forestry Sciences, Beijing, China; ^2^College of Information Technology, Shanghai Ocean University, Shanghai, China

**Keywords:** crack cottonseed identification, variational mode decomposition, sound to image encoding, vision transformer, deep learning, air-coupled ultrasound

## Abstract

Slight crack of cottonseed is a critical factor influencing the germination rate of cotton due to foamed acid or water entering cottonseed through testa. However, it is very difficult to detect cottonseed with slight crack using common non-destructive detection methods, such as machine vision, optical spectroscopy, and thermal imaging, because slight crack has little effect on morphology, chemical substances or temperature. By contrast, the acoustic method shows a sensitivity to fine structure defects and demonstrates potential application in seed detection. This paper presents a novel method to detect slightly cracked cottonseed using air-coupled ultrasound with a light-weight vision transformer (ViT) and a sound-to-image encoding method. The echo signal of air-coupled ultrasound from cottonseed is obtained by non-contact and non-destructive methods. The intrinsic mode functions (IMFs) of ultrasound signal are obtained as the sound features using variational mode decomposition (VMD) approach. Then the sound features are converted into colorful images by a color encoding method. This method uses different colored lines to represent the changes of different values of IMFs according to the specified encoding period. A light-weight MobileViT method is utilized to identify the slightly cracked cottonseeds using encoding colorful images corresponding to cottonseeds. The experimental results show an average overall recognition accuracy of 90.7% for slightly cracked cottonseed from normal cottonseed, which indicates that the proposed method is reliable to applications in detection task of cottonseed with slight crack.

## Introduction

Cotton is an important economic crop throughout the world. The quality of cottonseed is an important factor in determining the yield and quality of cotton. Cotton seed will go through a series of processes such as ginning and stripping, which will cause a lot of damage to cotton seed. However, in the process of removing excess linters of cottonseed, the foamed acid will enter the cottonseed through the cracks and diminish the germination of the cotton seed. After sowing, water will also enter cottonseed through cracks, further reducing the germination of cottonseed. Therefore, cracked cottonseeds will decrease cotton yield.

To reduce the amount of cottonseed wasted, the automation system of cotton precision seeding is often applied in practical production. After precision seeding, there is no need for thinning seedlings or avoiding inconsistency of individual growth and development in cotton field. Therefore, cotton precision seeding can significantly decrease the production cost of cotton, improve the efficiency of field management, and consequently, realize standardized planting. However, this technology puts forward higher requirements for the quality of cottonseeds, which makes the quality detection of cottonseeds crucial. The traditional seed detection method is destructive, inefficient, time-consuming, and non-automated. Developing fast and high-throughput non-destructive detection methods for seed quality is urgently needed for agricultural production. In recent years, non-destructive detection technologies, such as machine vision, optical spectroscopy, thermal imaging, and acoustics, have gradually become new ways to detect seed quality.

Machine vision is a rapid and non-destructive technology and has been applied to detect the quality and safety of seed. Based on morphological and color features extracted from images acquired by camera with high resolution, morphology (Rodríguez-Pulido et al., 2012), color (Tu et al., 2018), shape (Li et al., 2016), size, texture, and exterior defects (Huang et al., 2019) of seed can be evaluated. Severely damaged and broken cottonseeds can also be identified effectively by extracting morphological characteristics from image (Bai et al., 2018). However, injuries recognition and location are still major difficulties in using machine vision for seed surface defect detection (Huang et al., 2015). Slight injuries such as small cracks in cottonseed are hardly distinguished by vision. Moreover, it is particularly difficult for imaging the injuries on the edge and the back side of seed that are hidden from the camera's field of view.

Optical spectroscopy is also a powerful tool to inspect seed, especially in characterizing internal quality. Based on the interactions between light and material molecular groups, the information of corresponding chemical compositions can be extracted from the changes of optical spectra. According to the form of interaction, spectroscopic techniques are mainly based on light absorption (e.g., near-infrared spectroscopy), light scattering (e.g., Raman spectroscopy) and light emission (e.g., fluorescence spectroscopy). Most of them have been used to determine seed quality and safety, including internal compositions (Sunoj et al., 2016), moisture (Zhang and Guo, 2020), germination (Fan Y. et al., 2020) and infection (Tao et al., 2019). Hyperspectral imaging technique incorporates optical spectroscopy and imaging technology to obtain spatial and spectroscopic information simultaneously. Combined with machine learning methods, such as linear discriminant analysis (LDA), partial least-squares discriminant analysis (PLS-DA), support vector machine (SVM), and artificial neural networks (ANN), a hyperspectral imaging data cube can provide the information of chemical compositions and their distributions, which makes this a potential technology in the seed industry, especially in variety identification (Zhou et al., 2020), classification (Barboza da Silva et al., 2021) and chemical composition determination (Yang et al., 2018; Hu et al., 2021). Spectroscopic technology also does a good job in damage detection of seed, such as insect damage (Chelladurai et al., 2014), fungi damage (Baek et al., 2019), frost damage, and sprout damage, because all these types of damage can lead to the change of chemical compositions of seed, which could change the spectral features. Nevertheless, it is still challenging to distinguish pure physical damage with little chemical change, such as slight crack in cottonseed.

Thermal imaging is a non-destructive technique for converting the invisible infrared radiation pattern of an object into visible images for feature extraction and analysis (Rahman and Cho, 2016). Based on the changes of surface temperature, the infrared radiation profile of seed can be mapped and analyzed. Unlike above-mentioned methods, no illumination sources are required in this system, and a thermal imaging camera along with its data acquisition system is enough to provide information of object. Thermal imaging technology has found its way in estimating seed quality, including determination of morphological features, detection of diseases and insect infestation, evaluation of viability (Belin et al., 2018) and germination performance (Fang et al., 2016), distinguishing aged or dead seeds from healthy ones (Kim et al., 2014), and monitoring seed quality during storage (Xia et al., 2019). In addition, thermal imaging has the capability of sensing all possible physical damage of seed, since there is a significant relationship between seed temperature and degree of damage (ElMasry et al., 2020). But the difficulties of detecting slight physical injuries, which are hard to recognize by machine vision and even human vision, still exist for the thermal imaging method.

An acoustic method is also developed for non-destructive detection of agricultural products. Among acoustic technology, ultrasonic testing is an important method. With the advantages of short wavelength, high frequency, and good directional property, ultrasound possesses better penetrability than audible sound and subsonic wave, and consequently becomes a powerful tool in non-destructive testing of seed. Ultrasound signal produced by impacting seed can be used to evaluate seed quality. When crack appears on seed testa, the structural strength and damping coefficient of the seed will change, which leads to the variations of frequency and intensity of the impacting ultrasound signal. Depending on the differences in echo signals between healthy seed and defective seed, the acoustic method shows more superiority in recognizing fine surface crack than common non-destructive detection methods. The approach was first proposed to distinguish pistachio nuts with open shells from those with closed shells (Pearson, 2001), and the results showed that the detection accuracy of pistachio nuts was ~97%. Combined with signal processing and identifying algorithm, the acoustic method is applied to detect insect damage (Pearson et al., 2007; Yanyun et al., 2016) and mildew damage (Sun et al., 2018) of seed. The potential to identify fine defects with this method is expected and verified. However, detecting light crack of cottonseed with smaller size than most seeds is rarely reported.

In this paper, a non-destructive detection method based on an air-coupled ultrasonic inspection system is developed to distinguish cottonseed with slight crack from intact kernel. VMD is utilized to decompose an ultrasonic signal into band-limited multiple IMFs. These IMFs are used to construct the feature matrix of ultrasonic signal. Then the feature matrix is converted into the colorful image using a color encoding method. A deep learning-based method combining Transformer model with CNN model is used to classify the color images generated from air-coupled ultrasonic cottonseeds. Finally, the performance of the proposed method is compared with other detection methods.

## Materials and methods

### Samples

**Figure 1:**
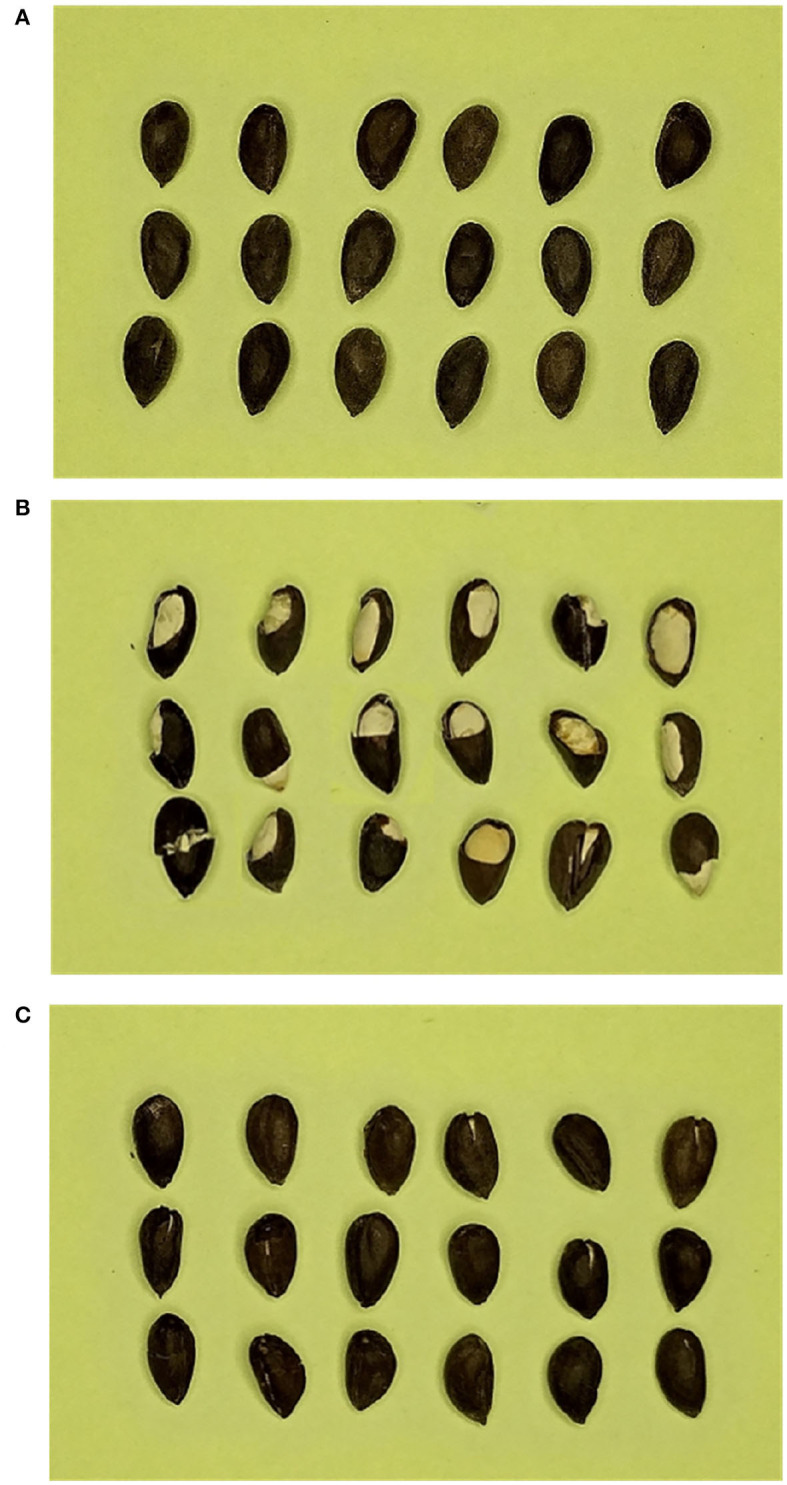
Cottonseed kernels. **(A)** Intact cottonseeds. **(B)** Cottonseeds with severe cracks. **(C)** Cottonseeds with slight cracks.

A total of 600 cottonseeds named Xinluzhong52 are used in this study. The total cottonseeds consisted of 296 intact kernels and 304 kernels with slight crack. Cracked cottonseed is damaged cottonseed showing the obvious white endosperm inside. For slightly cracked cottonseed, it is difficult to detect the endosperm inside, but there is crack on testa of cottonseed. Physical images of cottonseed are shown in Figure 1. The cottonseed with severe crack that shows the white endosperm can be identified by machine vision method. This research focuses on the method of distinguishing cottonseed with slight crack from intact cottonseed. Therefore, the samples only include the two classes of cottonseeds.

### Detection system based on air-coupled ultrasound

To maintain sufficient energy transmission, liquid couplant, such as water, oil etc. is used to immerse samples in a traditional ultrasonic technique. The mode of contact coupling may cause damage or pollution on the surface of sample. The air-coupled ultrasonic technique emerges as a novel approach for non-destructive, non-contact, and rapid inspection (Fang et al., 2017). The surrounding air is used as the couplant between transmitting transducer and materials or between receiving transducer and materials in air-coupled ultrasonic techniques. The significant advantage of an air-coupled ultrasonic technique is avoiding the use of traditional couplant. Therefore, it becomes a reliable and effective non-destructive detection method. The air-coupled ultrasonic technique is suitable for industrial detection applications, such as the natural defects in wood (Tiitta et al., 2020), corn seed with hole (Yanyun et al., 2016) and food engineering (Fariñas et al., 2021a,b).

**Figure 2:**
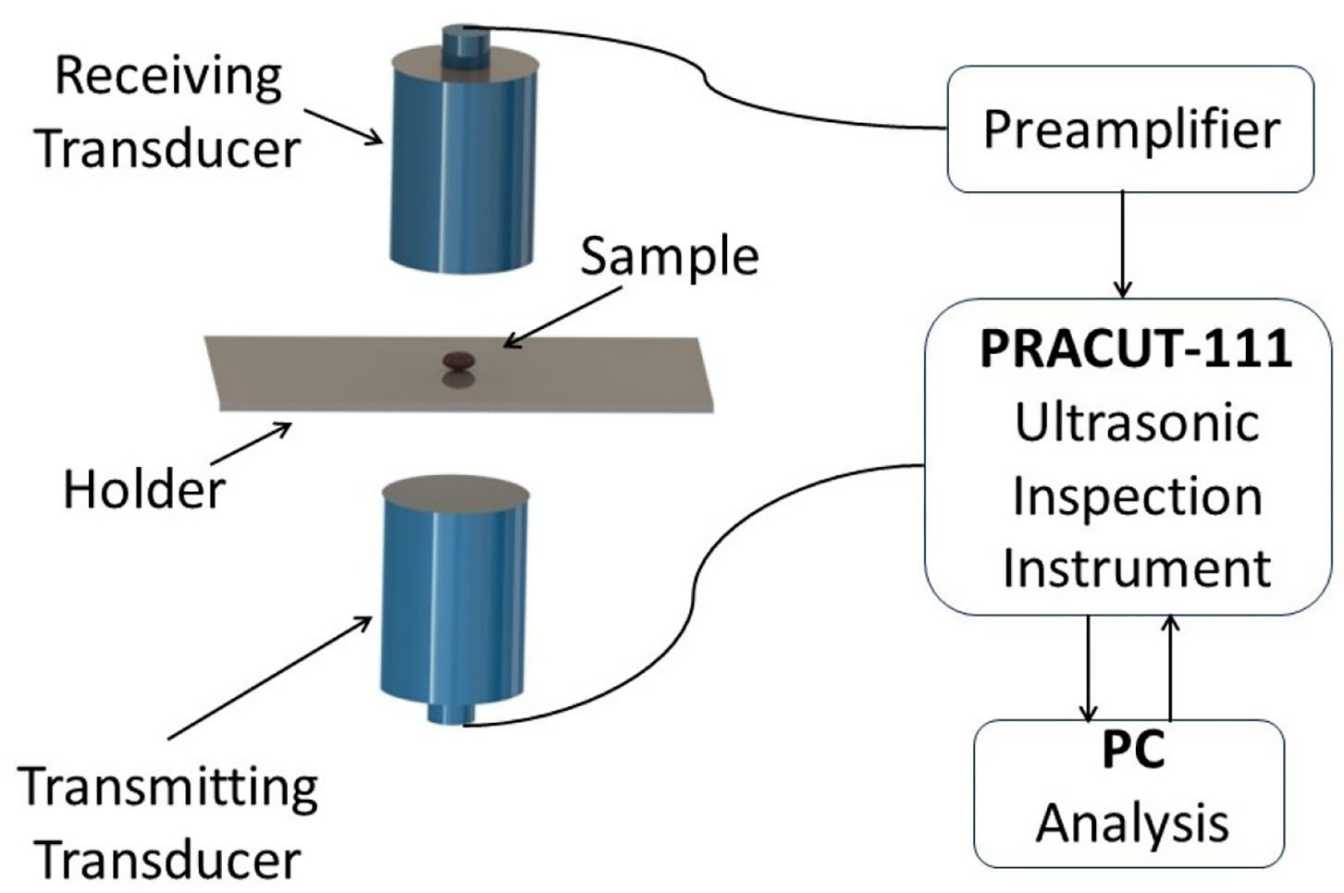
Scheme of air-coupled ultrasonic inspection system set-up.

The air-coupled ultrasonic detection system is used to obtain the ultrasonic echo signal. The inspection system is shown in Figure 2. The signal acquisition system consists of a pair of transducers (400K-20N-R50-T and 400K-20N-R50-R, PR, China), a preamplifier (400K, PR, China), an air-coupled ultrasonic inspection instrument (PRACUT-111, PR, China), and an industrial computer. The center frequency of the transducer is 400 *kHz*. The diameter of the piezoelectric ceramic disc is 20 mm. The focal length of the transducer is 50 mm. The normal through-transmission mode is applied to two transducers. In order to obtain accurate ultrasonic signals, the two transducers need to be strictly aligned. The cottonseed is placed as the focus between transmitting transducer and receiving transducer. To meet the requirement of cottonseed placement, an aluminum plate with a thickness of 0.2 mm as a holder is fixed in the middle of two transducers. The thin aluminum plate can guarantee that the air-coupled ultrasonic signal transmitted by the transmitting transducer can be received by the receiving transducer as much as possible. The preamplifier with the amplification factor of 60 dB is used to amplify and filter the ultrasonic signal received by the air-coupling receiver transducer (400K-20N-R50-R), and then input the processed ultrasonic signal to the air-coupled ultrasonic inspection instrument. The ultrasonic inspection instrument outputs 400 V excitation signal to drive the transmitting transducer and converts the received ultrasonic signal into digital signal. The industrial computer mainly consists of Intel i7-6700 CPU @3.4 GHz, 512G SSD hard drive, 16 GB RAM and a GPU (Geforce RTX 2080 WindForce OC 8G, GIGABYTE). The computer sends the control command to the ultrasonic inspection instrument through the USB interface, and receives the ultrasonic data sent by the ultrasonic inspection instrument through a LAN interface. The ultrasonic data are stored in the computer using PRACUT software (Suzhou Phaserise Technology Co., Ltd., China). Although Python language and Pytorch deep learning framework are used to realize the signal processing and the identification of cottonseed with slight crack, real-time detection can be realized by C++ language with the standard dynamic link library provided by PRACUT software.

### Ultrasonic signal acquisition

**Figure 3:**
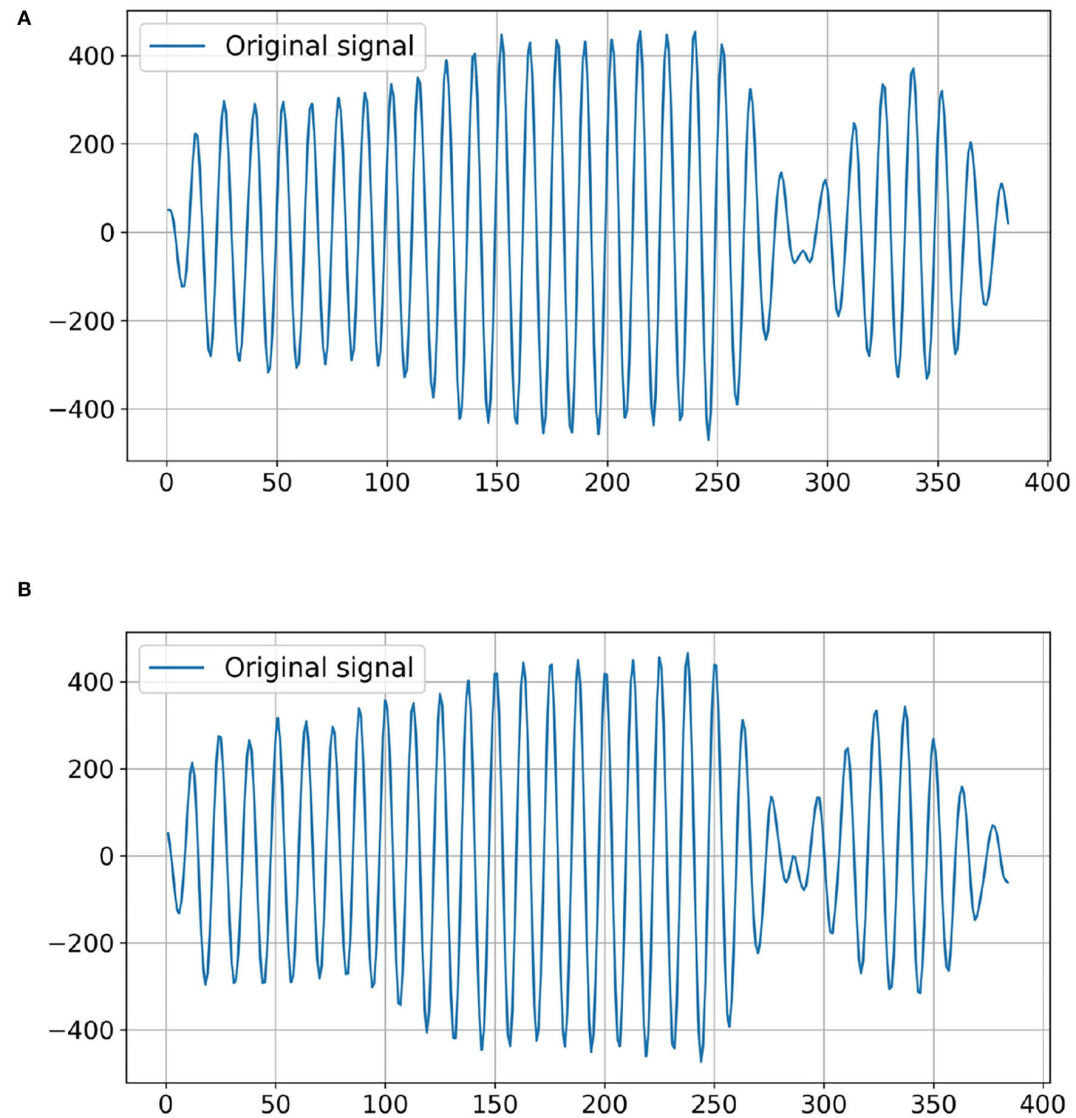
Examples of the original ultrasonic signals of cottonseed. **(A)** Intact cottonseed. **(B)** Cottonseed with slight crack.

A-scan mode of the air-coupled ultrasonic inspection instrument is used to identify cottonseed with slight crack. In A-scan mode, damaged cottonseeds are detected through changes in air-coupled ultrasonic signal passing through them. In order to generate the ultrasonic signal data set, first, the cottonseed sample is placed on the aluminum plate so that it coincides with the focus position of the transducers. Then the ultrasonic signal is obtained after the ultrasonic signal passes through the cottonseed. The ultrasonic signal data is exported and saved as a CSV format file. Finally, the category label (1 or 0) corresponding to each sample is appended to the end of CSV file, where “1” represents the normal cottonseed and “0” represents the cottonseed with slight crack. Each cottonseed sample corresponds to a CSV file, and all CSV files constitute the data set used in this study. The typical ultrasonic signals from intact cottonseed and cottonseed with slight crack are shown in Figure 3 respectively. The amplitude fluctuations of the two types of ultrasonic signals are very similar, so it is very important to extract effective features for classification from these signals.

### Identification of slight crack cottonseed

#### Variational mode decomposition method

Variational mode decomposition (VMD) (Dragomiretskiy and Zosso, 2013) is one of novel non-stationary signal decomposition techniques and has been recently applied in wind speed forecasting and many other fields (Dibaj et al., 2021; Yildiz et al., 2021). The original non-stationary signal can be decomposed into different band-limited intrinsic mode functions (IMFs) using VMD, similar to empirical mode decomposition (EMD). A non-recursive method is used to decompose original signal *X*(*t*) into *M* modes or subsequences *u*_*m*_(*m* = 1, 2, …, *M*). These IMFs have different central frequencies with finite bandwidths. The purpose of the transformation is to minimize the sum of the estimated frequency bandwidth of each IMF, and the constraint condition is that the sum of each IMF is equal to the input signal *X*(*t*). The objective function and constraint condition corresponding to the variational constraint model can be represented by the following Equation 1:


(1) $$\{\begin{array}{c} \underset{\left\{u_{m}\right\},\left\{\omega_{m}\right\}}{\min}\overset{M}{\underset{m=1}{\sum }}{\left\|ɓ\left[\left(\delta \left(t\right)+\frac{j}{\pi t}\right)*u_{m}(t)e^{-j\omega_{k}t}\right]\right\|}_{2}^{2}\\ s.t.\;X\left(t\right)=\sum_{m=1}^{M}u_{m} \end{array}$$

where δ(*t*) is Dirac function; * denotes the convolution operation in signal processing; *j* is an imaginary number; || · || denotes the *L*^2^- norm; {*u*_*m*_} = {*u*_1_, *u*_2_, …, *u*_*M*_} is the set of all IMFs; {ω_*m*_} = {ω_1_, ω_2_, …, ω_*M*_} is the set of central frequencies corresponding to all IMFs.

In order to resolve the constrained variational optimization problem, quadratic penalty factor term α and Lagrange multipliers λ(*t*) are defined, so the optimization problem can be transformed into an unconstrained variational problem. The constructed unconstrained form can be presented in Equation 2.


(2) $$\begin{array}{l} L(\left\{u_{m}\right\},\left\{\omega_{m}\right\},\left\{\lambda \left(t\right)\right\}\\ =\alpha \sum_{m=1}^{M}{\left\|ɓ\left[\left(\delta \left(t\right)+\frac{j}{\pi t}\right)*u_{m}\left(t\right)e^{-j\omega_{k}t}\right]\right\|}_{2}^{2}\\ +{\left\|X\left(t\right)-\sum_{m=1}^{M}u_{m}\right\|}_{2}^{2}+\langle \left\{\lambda \left(t\right)\right\},X\left(t\right)-\sum_{m=1}^{M}u_{m}\rangle \end{array}$$

where α is used to ensure high reconstruction fidelity even in the presence of additive Gaussian white noise; λ(*t*) is used to strictly ensure the constraints.

To solve the unconstrained optimization problem in Equation 2, the alternating direction multiplier method (ADMM) (Hong and Luo, 2017) is used to find the saddle point of the optimization problem. The minimax point of the augmented Lagrangian function L can be obtained by updating *u*_*m*_, ω_*m*_ and λ alternately. The iteration process of *u*_*m*_, ω_*m*_ and λ are as following Equation 3, 4, 5:


(3) $$\begin{array}{l} {û}_{m}^{n+1}\left(\omega \right)=\frac{\hat{f}\left(\omega \right)-\sum_{i\neq m}{û}_{i}\left(\omega \right)+\frac{\hat{\lambda }\left(\omega \right)}{2}}{1+2\alpha {\left(\omega {-\;\omega }_{m}\right)}^{2}} \end{array}$$

(4) $$\begin{array}{l} \omega_{m}^{n+1}=\frac{\int_{0}^{\infty }\omega |{û}_{m}\left(\omega \right){|}^{2}d\omega }{\int_{0}^{\infty }|{û}_{m}\left(\omega \right)\;{|}^{2}d\omega } \end{array}$$

(5) $$\begin{array}{l} {\hat{\lambda }}^{n+1}\left(\omega \right)={\hat{\lambda }}^{n}\left(\omega \right)+\tau \left(\hat{X}\left(\omega \right)-\underset{m}{\sum }{û}_{m}^{n+1}\left(\omega \;\right)\right) \end{array}$$

where *n* is the number of iterations; $\hat{X}\left(\omega \right)$, ${û}_{m}^{n+1}\left(\omega \right)$, û_*i*_(ω) and $\hat{\lambda }\left(\omega \right)$ can be obtained from *X*(*t*), $u_{m}^{n+1}\left(t\right)$, *u*_*i*_(*t*) and λ(*t*) by the Fourier transforms. If the convergence condition shown in Equation 6 is satisfied, the iteration will be terminated. Finally, through the inverse Fourier transform of ${û}_{m}^{n+1}\left(t\right)$, the real part of the result is taken as the mode functions $u_{m}^{n+1}\left(t\;\right)$.


(6) $$\begin{array}{l} \overset{M}{\underset{m=1}{\sum }}\frac{||{û}_{m}^{n+1}-{û}_{m}^{n}|{|}_{2}^{2}}{||{û}_{m}^{n}|{|}_{2}^{2}}<\;\epsilon \end{array}$$

The optimization process for VMD is as follows:

Step 1: Initialize $u_{m}^{1}$, $\omega_{m}^{1}$, λ^1^ and *n*, where *n* = 1.
Step 2: Set *n* to *n* + 1 and update ${û}_{m}^{n+1}\left(\omega \right)$, $\omega_{m}^{n+1}$ and ${\hat{\lambda }}^{n+1}$ according to Equation 3, 4, 5.
Step 3: Repeat step 2 until the iteration convergence condition in Equation 6 is satisfied. M narrowband IMF $u_{m}^{n+1}\left(t\right)$ can be obtained by using inverse Fourier transform.

**Figure 4:**
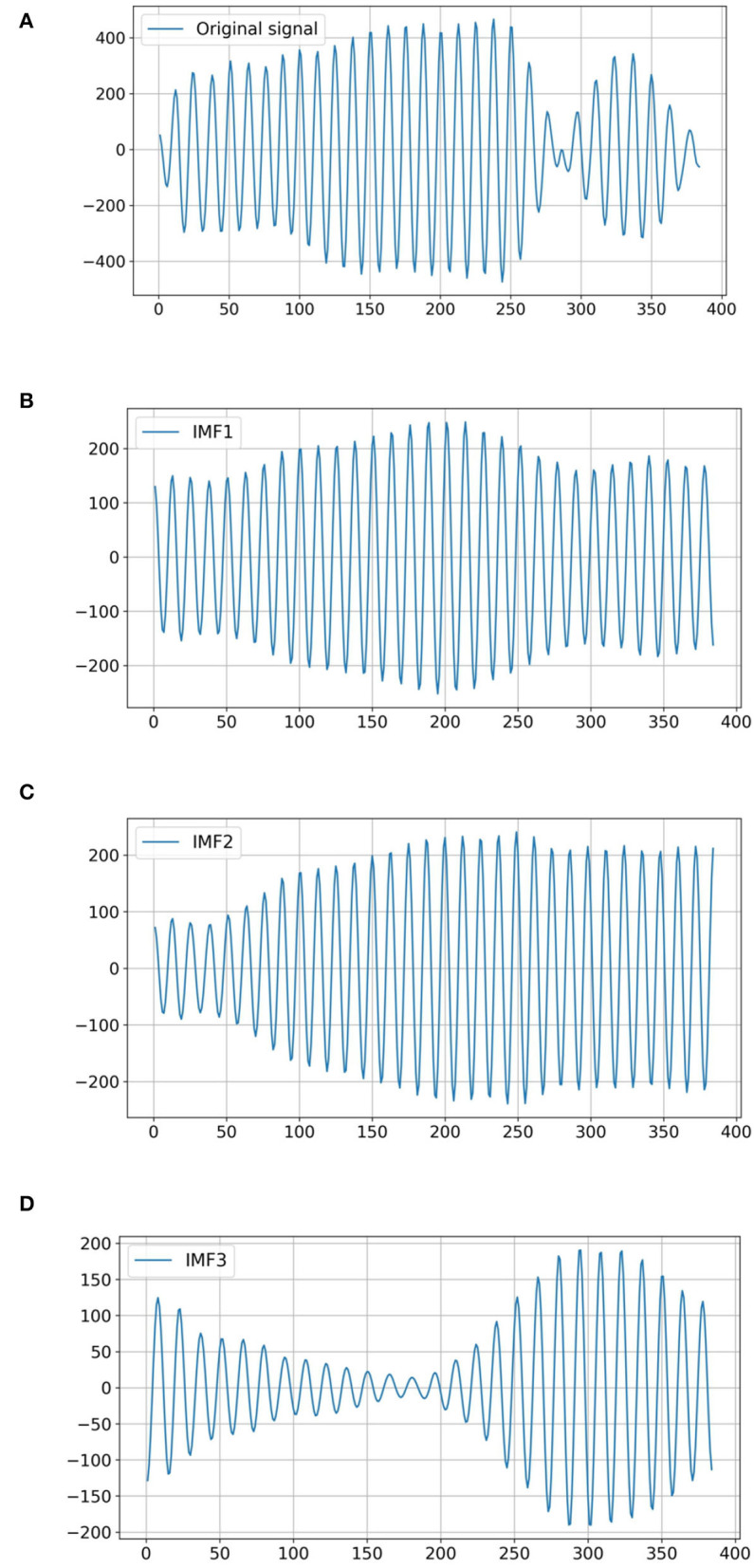
The VMD decomposition from the air-coupled ultrasonic signal of a cottonseed with slight crack. **(A)** Original signal. **(B)** IMF1. **(C)** IMF2. **(D)** IMF3.

The air-coupled ultrasonic signal of a cottonseed with slight crack is used as the examples for VMD decomposition. The results of VMD decomposition are shown in Figure 4. Because of the similarity between air-coupled ultrasonic signal of intact cottonseed and that of cottonseed with slight crack, it is important to extract the air-coupled ultrasonic signal features from these intrinsic mode functions.

#### Encoding from ultrasound to image

The ultrasonic signal *X*(*n*) acquired from air-coupled ultrasonic detection system are decomposed to *M* intrinsic mode functions *u*_*m*_, *m* ϵ {1, 2, …, *M*} using VMD transformation. *M* IMFs are stacked to generate the intrinsic mode functions matrix *S* of air-coupled ultrasonic signal *X*(*n*) according to Equation 7:


(7) $$\begin{array}{l} S=\left[\begin{array}{c} u_{1}\\ u_{2}\\ \vdots \\ u_{M} \end{array}\right]=\left[s_{1},s_{2},\cdots \;,s_{L}\;\right] \end{array}$$

where *S* ∈ ℝ^*M*×*L*^ and *L* is the length of the air-coupled ultrasonic signal *X*(*n*). *u*_*m*_ is composed of *L* discrete values with the same length as the ultrasonic signal, which can be represented as $u_{m}\in {\mathbb{R}}^{1\times L}$. Vector *s*_*l*_ is composed of *M* discrete values in the column direction of the matrix *S* which can be represented as $s_{l}\in {\mathbb{R}}^{M\times 1}$. In order to convert the ultrasonic signal *X*(*n*) into a color-coded image *I*_*C*_, the color set *C* is defined for color coding, *C* = {*c*_1_, *c*_2_, ⋯ , *c*_*B*_}, where *c*_*i*_ represents different colors and *B* is the number of different colors in the color set *C*. First, vector *s*_1_ at the first column of the intrinsic mode functions matrix *S* is selected and converted into a part of colorful image *I*_*C*_. Here, *s*_1_ = {*u*_11_, *u*_12_, ⋯ , *u*_1*M*_}. Color *c*_1_ in set *C* is chosen and used to draw a polyline in the image *I*_*C*_ according to the value of *s*_1_. Then vector *s*_2_ at the second column of matrix *S* and color *c*_2_ are selected to draw the second polyline in the image *I*_*C*_ to represent *s*_2_. According to this method, color *c*_1_ is used to draw the next polyline again after completing the drawing of *B* colorful polylines. Finally, *B* is considered as the cycle to complete the drawing of *L* colorful polylines. Image *I*_*C*_ is generated from the air-coupled ultrasonic signal *X*(*n*) using the above color encoding method.

**Figure 5:**
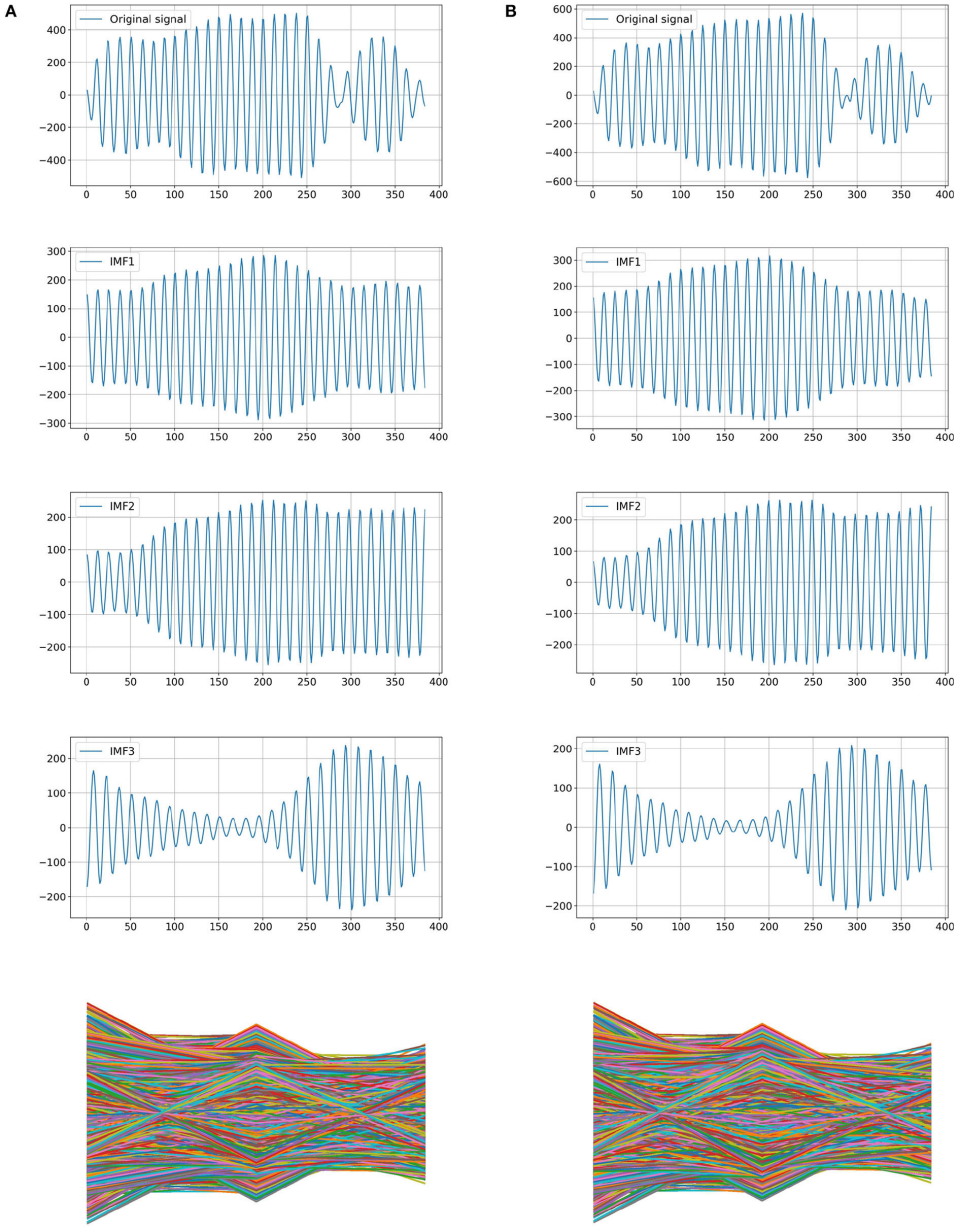
Colorful images generated from the ultrasonic signals. **(A)** The process of generating image from the ultrasonic signal of intact cottonseed. **(B)** The process of generating image from the ultrasonic signal of slightly cracked cottonseed.

In order to accurately convert the ultrasonic signal into a colorful image, first, the drawing ranges *W*_*S*_, *H*_*S*_ in image *I*_*C*_ and the coordinates (*x*_*O*_*S*__, *y*_*O*_*S*__)of the image origin *O*_*S*_ are defined. Then the coordinates required to draw polylines are calculated according to Equation 8:


(8) $$\{\begin{array}{c} x_{s_{lm}}=x_{O_{S}}+\frac{(m-1)W_{S}}{M-1}\;m\in \left\{1,2,\cdots ,M\right\}\\ y_{s_{lm}}=y_{O_{S}}+\frac{\left(H_{S}-y_{O_{S}})(S_{\max}-S_{lm}\right)}{\left(S_{\max}-S_{\min}\right)}\;l\in \left\{1,2,\cdots ,L\right\} \end{array}$$

where *S*_max_ and *S*_min_ are respectively the maximum and minimum values in the matrix *S* composed of *M* intrinsic mode functions after VMD decomposition of each ultrasonic signal. The position of each column vector of matrix *S* in the image *I*_*C*_ is determined by Equation 8, and then the corresponding position is connected with the same color to complete the drawing of polylines. For each vector *s*_*l*_, *M –* 1 colorful lines need to be drawn. In this study, let *M* = 3, *L* = 450, *B* = 10, the size of the generated image *I*_*C*_ is 1,800 × 1,200 and the origin coordinate of the encoding image part on image *I*_*C*_ is (288,1010). The process of generating images from the ultrasonic signals of intact cottonseed and slight cracked cottonseed is shown in Figure 5. As seen in Figure 5, the coding representation of ultrasonic signals from one-dimensional ultrasonic signal to two-dimensional images can be realized by polylines with different colors according to the results of VMD decomposition of ultrasonic signals.

In practical application, in order to obtain the optimal detection effect, the air-coupled ultrasonic data of 30 intact cottonseeds and 30 cottonseeds with slight crack are randomly selected from the air-coupled ultrasonic data set, the background image is generated according to the above method, and then the air-coupled ultrasonic data of the remaining 540 cottonseeds are used to generate colorful encoding images on the background image. So, the conversion from sound to image is completed. Finally, the image data set including 266 images generated from intact kernels and 274 images generated from slight crack kernels is obtained. In this study, 80% of the data set is used to train the model and 20% is used to test the effect of the training model.

#### MobileVIT vision transformer model

The ransformer-based model achieves great success in the natural language processing (NLP) field (Vaswani et al., 2017). Transformer has a layer stacking architecture, which only uses the multi-Head self-attention mechanism without convolution and recursion. Inspired by the successful application of transformer in NLP field, Dosovitskiy et al. (2020) propose Vision Transformer (ViT) model which employed a standard Transformer directly to visual tasks. In this method, the image is divided into a sequence of patches, and the linear embedding sequence of these image patches are taken as the input of the Transformer. The processing method of image patches is the same as that of tokens in NLP application, and excellent results are achieved on massive data sets. Liu et al. (2021), proposed the Swin Transformer (Shifted Window Transformer) model that could replace the classic convolutional neural network (CNN) architecture and become a general backbone in the field of computer vision. This model is based on the idea of ViT model and shows the effectiveness on different vision problems using patch merging and the shifted window with self-attention.

**Figure 6:**
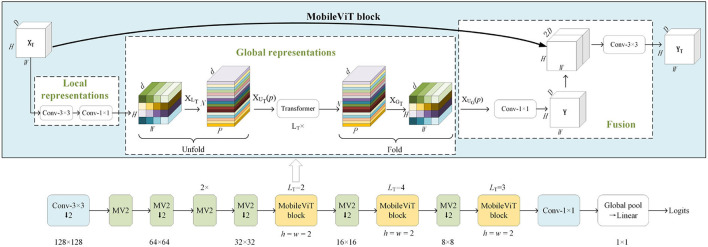
The architecture of Mobile ViT network.

Although Vision Transformer-based model can be an alternative to CNNs in computer vision field, the large model size, high requirement for training data, and latency of Vision Transformer limits its practice application, especially for resource constrained equipment. In order to obtain a lightweight and efficient architecture of Vision Transformer model, MobileViT (Mehta and Rastegari, 2021) is proposed for mobile vision applications. MobileViT combines the advantages of transformers and convolutions, so it can encode the local information obtained from convolutions and global information obtained from transformers in tensors without lacking in inductive bias. The model structure of MobileViT is shown in Figure 6, where MV2 block represents MobileNetV2 block with inverted residual structure. ↓2 refers to down-sampling operation. A standard 3 × 3 convolution operation is represented by *Conv*-3 × 3 block in MobileViT model. MobileViT block combines convolutions with transformers to learn the global and local information from input tensor respectively. Tensor $X_{T}\in {\mathbb{R}}^{H\times W\times D}$passes through a series of convolution operations and tensor $X_{L_{T}}\in {\mathbb{R}}^{H\times W\times d}$ is obtained. Then tensor *X*_*L*_*T*__ is divided into *N* patches with width *w* and height *h* and $X_{U_{T}}\in {\mathbb{R}}^{P\times N\times d}$ can be obtained by unfolding *X*_*L*_*T*__. For example, the *X*_*L*_*T*__ in Figure 6 is equally divided into 4 × 4 small patches, then a d-dimensional vector is extracted at the same position of each patch. The corresponding vectors from the same position *p* ∈ {1, ⋯ , *P*} are combined and used to generate tokens. The inter-path relationships $X_{G_{T}}\in {\mathbb{R}}^{P\times N\times d}$ can be obtained by encoding operation with Transformers as Equation 9:


(9) $$\begin{array}{l} X_{G_{T}}=Transformer\left(X_{U_{T}}\left(p\right)\right),1\leq p\leq P \end{array}$$

where local information can be encoded by *X*_*U*_*T*__(*p*) and global information passing through *p*-*th* position in *P* patches can be encoded by *X*_*U*_*G*__(*p*). The output *Y*_*T*_ of MobileViT block can be obtained after convolution and concatenation operations.

The computation of self-attention in above Transformer is realized using scale dot-product attention according to Equation 10:


(10) $$\begin{array}{l} Attention\left(Q,K,V\right)=SoftMax\left(\frac{QK^{T}}{\sqrt{d_{k}}}\right)V \end{array}$$

where *Q, K* and *V* represent the query, key, and value matrices respectively. *d*_*k*_ refers to the dimension of *Q* and *K*. The *SoftMax* function is used to obtain the weights of *V*. Spatial order of pixels will be ignored in standard ViTs. But both the spatial order of pixels and the path order will be obtained in MobileViT model.

## Results and discussion

### Cottonseed germination test

It is important to guarantee the safety of air-coupled ultrasonic detection for cottonseed. In order to verify the safety of ultrasonic detection, the germination test of cottonseeds is used as the verification method. First, two batches of intact cottonseeds are randomly selected, then the cottonseeds are divided into two groups. One group is not used to ultrasonic testing, and the other group is passed through air-coupled ultrasonic at the same frequency and intensity in detection experiment. For each germination test, 1,000 cottonseeds after ultrasonic testing and 1,000 cottonseeds without ultrasonic testing are selected respectively. Then the two groups of cottonseeds are used in cottonseed germination tests at the same time. It is determined whether air-coupled ultrasonic detection will cause damage to the cottonseeds by the comparison of the germination rates.

Table 1 shows the comparative results of the cottonseed germination rate. By comparing the results in Table 1, it can be seen that air-coupled ultrasonic detection does not affect the germination rate of cottonseeds. At the same time, the germination rate of seeds tends to decrease with the increase of time. This trend may be due to the storage environment of cottonseeds not in accordance with the storage standards, which affects the germination rate of cottonseeds. Therefore, in this study, the air-coupled ultrasound with frequency of 400 kHz and voltage of 200 V is used to detect the slight crack of cottonseed, which will not damage the cottonseeds or reduce the germination rate of cottonseeds. This is a safe non-destructive detection method.

**Table 1:** The results of cottonseeds germination test.

**Cottonseed**	**Storage for**	**Storage**
**category**	**2 months**	**1 year**
Cottonseeds after ultrasonic testing	83.5%	72.2%
Cottonseeds without ultrasonic testing	86.1%	70.6%

### Determination of decomposition number *M* using sample entropy

The ultrasonic signal *X*(*n*) is decomposed to *M* intrinsic mode functions using VMD decomposition. The number *M* of intrinsic mode functions needs to be determined artificially. Sample entropy is proposed to measure the complexity of time series by Richman and Moorman (2000). Complex time series signals have large sample entropy, while time series signals with strong selfsimilarity have small sample entropy. Zhang et al. (2020) proposed a method to select the number *M* of intrinsic mode functions by computing the sample entropy of time series. For an appropriate *M* value of VMD decomposition operation, it will correspond to smaller sample entropy of the time sequence signal. Therefore, on the condition of effectively limiting the computational complexity, the *M* value is determined by calculating the sample entropy of the intrinsic mode functions.

For one-dimensional time series signal {*X*_*SE*_(*i*), *i* = 1, 2, …, *L*}, *z*-dimensional vector is reconstructed and represented by {*Y*_*SE*_(*i*), *i* = 1, 2, …, *Z, Z* = *L – z* + 1} according to Equation 11:


(11) $$\begin{array}{l} Y_{SE}\left(i\right)=\{X_{SE}\left(i\right),X_{SE}\left(i+1\right),X_{SE}\left(i+2\right),\cdots ,\\ \;X_{SE}\left(i+m-1\right)\} \end{array}$$

Then the maximum value of Euclidean distance between any component of vectors *Y*_*SE*_(*i*) and *Y*_*SE*_(*j*) is calculated according to Equation 12 and represented as *D*_*SE*_(*Y*_*SE*_(*i*), *Y*_*SE*_(*j*)).


(12) $$\begin{array}{l} D_{SE}\left(Y_{SE}\left(i\right),Y_{SE}\left(j\right)\right)=\max\left[|Y_{SE}\left(i+k\right)-Y_{SE}\left(j+k\right)|\;\right] \end{array}$$

where *i, j* ϵ {1, 2, …, *Z – z* + 1} and *k* ϵ {0, 1, …, *z* – 1}. Then $A_{i}^{z}\left(r\right)$ is calculated according to Equation 13, where *r* is tolerance threshold and $A_{i}^{z}$ is the number that the distance between *Y*_*SE*_ (*i*) and *Y*_*SE*_(*i*) is not greater than *r*.


(13) $$\begin{array}{l} {\bar{A}}_{i}^{z}\left(r\right)=\frac{1}{L-z+1}A_{i}^{z} \end{array}$$

Then $A_{i}^{z}\left(r\right)$ is used to calculate ${\bar{A}}_{i}^{z}\left(r\right)\cdot {\bar{A}}_{i}^{z}\left(r\right)=\frac{1}{L-z}\overset{L-z}{\underset{i=1}{\sum }}A_{i}^{z}\left(r\right)$. Finally, the sample entropy of time series signal is calculated according to the following to Equation 14:


(14) $$\begin{array}{l} SampleEn\left(m,r\right)=-\ln\frac{A_{i}^{z+1}\left(r\right)}{A_{i}^{z}\left(r\;\right)} \end{array}$$

**Figure 7:**
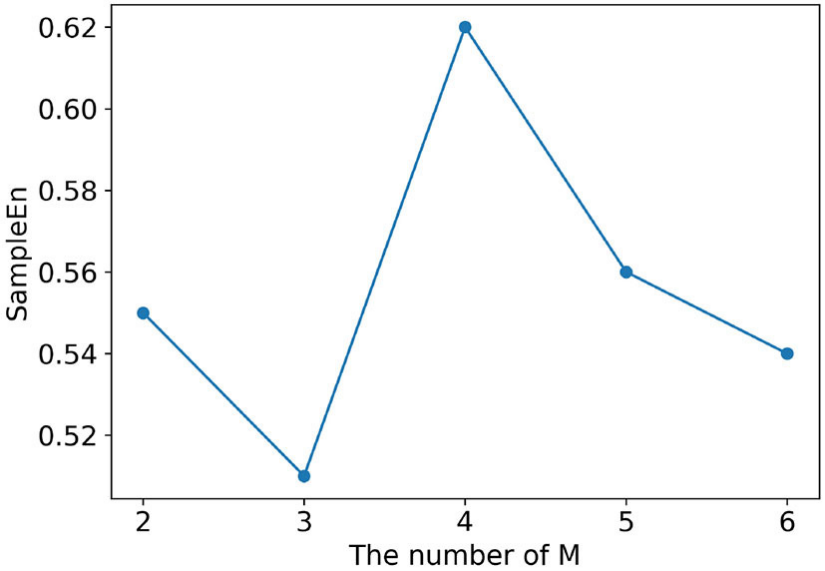
The value of the sample entropy with different numbers M of IMFs.

The original air-coupled ultrasonic signal is decomposed using VMD with different parameter *M*. Then the sample entropy corresponding to each *M* is calculated accordance to Equation 14. In order to obtain an appropriate detection speed, *M* is set to 2, 3, 4, 5, 6 in the experiment respectively. It can be seen from Figure 7 that the sample entropy has the smallest value when *M* is equal to 3. Therefore, *M* = 3 is determined as the number of intrinsic mode functions of VMD decomposition in this study.

### Influence of the number of encoding colors

**Figure 8:**
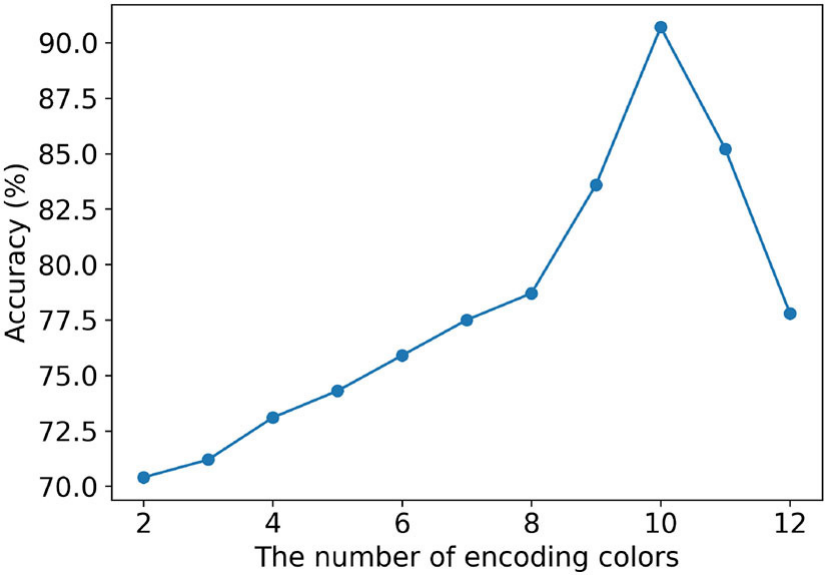
Accuracy of slight crack detection of cottonseed with different numbers of encoding colors.

The conversion from air-coupled ultrasonic data to image data is realized through a specific number of color encoding sets. In order to determine the optimal number of encoding colors for ultrasound to image conversion, a color set for encoding including 12 colors is constructed. The air-coupled ultrasonic signal decomposed by VMD method is encoded by two to 12 colors respectively. Eleven image data sets are constructed using the original air-coupled ultrasonic data. The MobileViT model is trained by using each image data set. The training data sets and test data sets from 11 image data sets are divided in the same proportion respectively. The initialization parameters of MobileViT in this study are shown in Table 2. These initialization parameters are mainly determined according to GPU performance and characteristics of encoding image. The comparison results of the MobileViT model obtained on the test sets of colorful images generated by different numbers of encoding colors are shown in Figure 8. The colorful image sets for tests come from the same air-coupled ultrasonic data test set, but different test sets are generated according to the corresponding color encoding method. It can be seen from Figure 8 that with the increase of encoding colors, the classification accuracy increases gradually. When the number of encoding colors is equal to 10, the classification accuracy reaches the maximum. Then the classification accuracy decreases with the increase of the encoding color number. This is mainly because, with the increase of the encoding colors number and the types of colors, the image generated from the air-coupled ultrasound becomes more complex. It is difficult for the classification algorithm to extract effective features from the complex image. Therefore, to realize the conversion from sound to image, 10 colors are selected to encode the intrinsic mode functions of the original air-coupled ultrasonic signal.

**Table 2:** Parameters of MobileViT for cottonseed with slight crack.

**Parameters**	**Value**
Input size	256 × 256
Classes	2
Batch size	16
Learning rate	1.0 × 10^−3^
Iterations	10

### Comparison of different methods

In order to compare the detection effects of the method proposed in this study with those of other methods, eight detection methods are proposed for the comparison experiment. The long short-term memory (LSTM) network is a special kind of recurrent neural network that can store and retrieve information from sequence data by memory cells (Hochreiter and Schmidhuber, 1997). LSTM network is an effective classifier for dealing with one-dimensional time-series and sequential data. The original air-coupled ultrasonic signal, wavelet features, and the results of VMD decomposition are used as input data respectively, and then combined with LSTM classifier to obtain four methods for the comparison. The wavelet transformer can be used to obtain the time-frequency domain features of original air-coupled ultrasonic signal.

**Figure 9:**
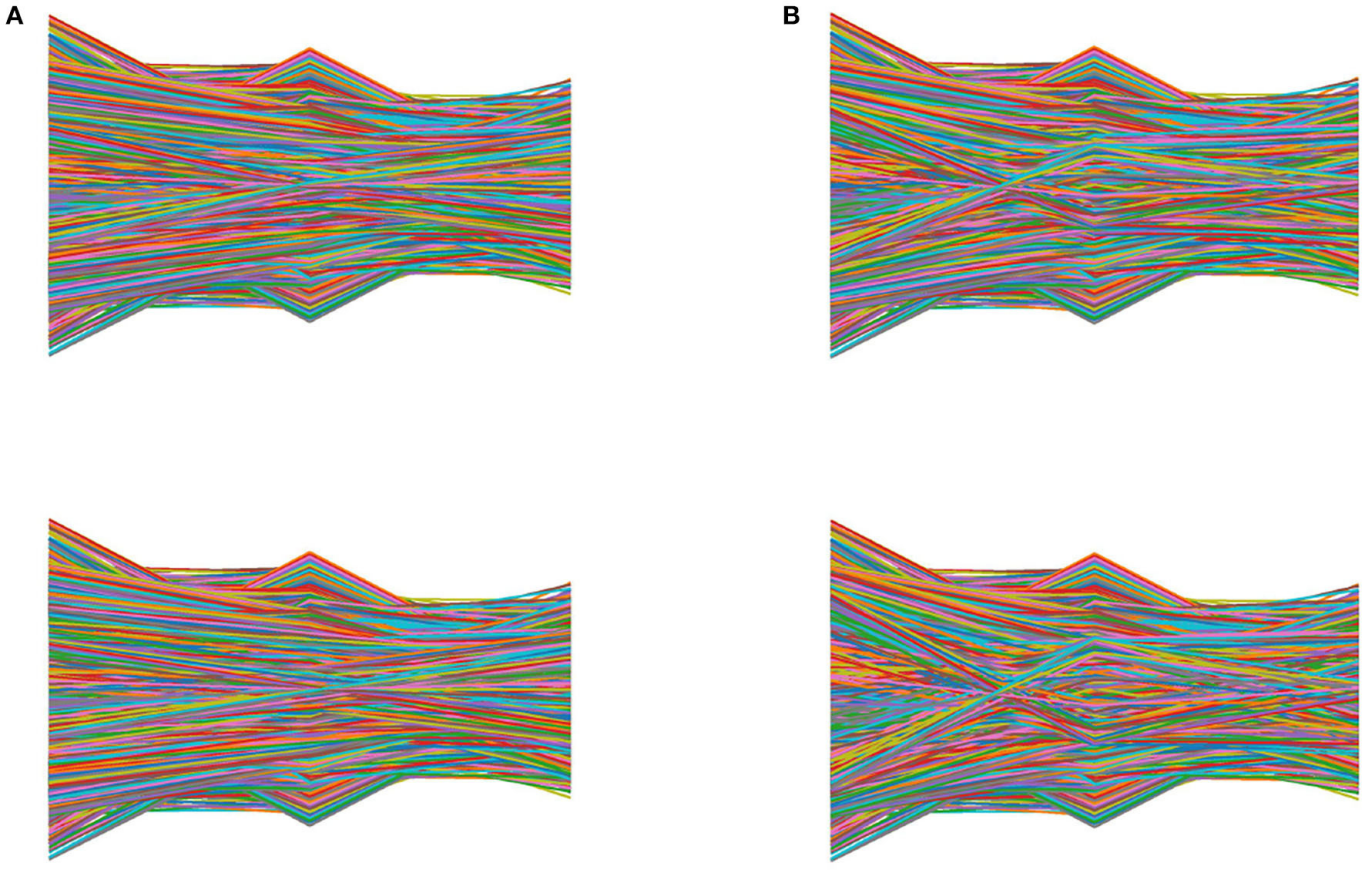
Color images generated from air-coupled ultrasound of cottonseeds. **(A)** Intact cottonseeds. **(B)** Cottonseeds with slight cracks.

The color images used to train deep learning classifiers are shown in Figure 9. For the data set obtained by the color encoding (CE) method from sound to image, different classifiers are used to compare the detection of cottonseed with slight crack. The traditional convolutional neural network (CNN) (Fan S. et al., 2020), residual network (ResNet18) (He et al., 2016) and Swin Transformer (Liu et al., 2021) are chosen as the classifier for the comparison respectively. The shortcut connections in ResNet18 make deeper neural networks to realize complex classification tasks. These models use a cross-entropy function as a loss function. Meanwhile, the training iterations of all models is set to 10.

The experimental results are shown in Table 3. From the experimental results, it can be seen that the two-dimensional image can better reflect the features of air-coupled ultrasonic signal than the features of one-dimensional signal. Because the MobileVIT model can combine the local analysis ability of convolutional neural network with the global analysis ability of Transformer, the classification performance of MobileViT is better than that of convolutional neural network and Swin Transformer model. Due to the vast amount of parameters in the Swin Transformer model and the requirement for a large number of training data, the training and learning of non-large data sets can't show the advantages of the Swin Transformer model. Therefore, VMD-CE–MobileViT approach proposed in this study can distinguish normal cottonseed from cottonseed with slight crack effectively.

**Table 3:** The comparison of different methods for the detection of cottonseed with slight crack.

**Method**	**Precision**	**Recall**	**F1 score**	**Accuracy**
1D raw data - LSTM	80%	65.5%	72%	74%
Wavelet - LSTM	64.1%	92.6%	78.5%	70.4%
VMD- LSTM	75.5%	68.5%	71.8%	73.1%
VMD- CE - CNN	94.4%	61.8%	74.7%	78.7%
VMD- CE - ResNet18	82.8%	96.4%	89.1%	88%
VMD- CE – Swin Transformer	73.1%	89.1%	80.3%	77.8%
VMD- CE – MobileViT (proposed method)	86.9%	96.4%	91.4%	90.7%

## Conclusion

In this paper, a detection method based on air-coupled ultrasound and sound to image encoding is proposed for slight crack identification of cottonseed. The traditional ultrasound detection method is not suitable for the requirements of non-destructive detection of cottonseed quality, and it is very difficult to detect cottonseed with slight crack using machine vision and other non-destructive detection technologies. To distinguish kernels with slight crack from intact kernels, a non-destructive, non-contact detection method based on air-coupled ultrasound is developed. VMD decomposition is used to obtain the IMFs of air-coupled ultrasonic signal. Then the feature matrix from the IMFs is applied to generate colorful image by a color encoding method. This method of converting sound into image can help the MobileViT classifier to obtain higher detection accuracy. The experimental results show that slightly cracked cottonseed can be distinguished from normal cottonseed precisely. The average accuracy of slightly cracked cottonseed identification test is 90.7%. The presented method can be extended to other signal recognition domain, such as distinguishing premature heartbeat signal from normal heartbeat signal in electrocardiogram (ECG) domain. In the future, we will combine the method proposed in this study with hyperspectral image processing technology to improve the detection accuracy further.

## Data availability statement

The original contributions presented in the study are included in the article/supplementary material, further inquiries can be directed to the corresponding authors.

## Author contributions

CZ proposed the idea, designed the methodology and prepared the manuscript. WH managed and coordinated the research activity planning and execution. XL conducted the experiment and maintained the research data. XH designed the experimental system. XT assisted in analyzing experimental data. LC provided oversight and leadership responsibility for the research. QW reviewed, edited the manuscript, and is responsible for ensuring that the descriptions are accurate and agreed by all authors. All authors contributed to the article and approved the submitted version.

## Funding

This work was supported by the National Natural Science Foundation of China (NSFC No. 31871523), Young Elite Scientists Sponsorship Program by CAST (2019QNRC001), and National Natural Science Foundation of China (NSFC No. 31901402).

## Conflict of interest

The authors declare that the research was conducted in the absence of any commercial or financial relationships that could be construed as a potential conflict of interest.

## Publisher's note

All claims expressed in this article are solely those of the authors and do not necessarily represent those of their affiliated organizations, or those of the publisher, the editors and the reviewers. Any product that may be evaluated in this article, or claim that may be made by its manufacturer, is not guaranteed or endorsed by the publisher.
